# A computer-aided system improves the performance of endoscopists in detecting colorectal polyps: a multi-center, randomized controlled trial

**DOI:** 10.3389/fmed.2023.1341259

**Published:** 2024-01-24

**Authors:** Heng Zhang, Qi Wu, Jing Sun, Jing Wang, Lei Zhou, Wei Cai, Duowu Zou

**Affiliations:** ^1^Department of Gastroenterology, The Central Hospital of Wuhan, Tongji Medical College, Huazhong University of Science and Technology, Wuhan, China; ^2^Endoscopy Center, Peking University Cancer Hospital and Institute, Beijing, China; ^3^Department of Gastroenterology, Ruijin Hospital, Shanghai Jiao Tong University School of Medicine, Shanghai, China; ^4^Department of Gastrointestinal Surgery, The Central Hospital of Wuhan, Tongji Medical College, Huazhong University of Science and Technology, Wuhan, China

**Keywords:** computer-aided detection, artificial intelligence, colorectal polyps, colonoscopy, sensitivity

## Abstract

**Background:**

Up to 45.9% of polyps are missed during colonoscopy, which is the major cause of post-colonoscopy colorectal cancer (CRC). Computer-aided detection (CADe) techniques based on deep learning might improve endoscopists’ performance in detecting polyps. We aimed to evaluate the effectiveness of the CADe system in assisting endoscopists in a real-world clinical setting.

**Methods:**

The CADe system was trained to detect colorectal polyps, recognize the ileocecal region, and monitor the speed of withdrawal during colonoscopy in real-time. Between 17 January 2021 and 16 July 2021. We recruited consecutive patients aged 18–75 years from three centers in China. We randomized patients in 1:1 groups to either colonoscopy with the CADe system or unassisted (control). The primary outcomes were the sensitivity and specificity of the endoscopists. We used subgroup analysis to examine the polyp detection rate (PDR) and the miss detection rate of endoscopists.

**Results:**

A total of 1293 patients were included. The sensitivity of the endoscopists in the experimental group was significantly higher than that of the control group (84.97 vs. 72.07%, *p* < 0.001), and the specificity of the endoscopists in these two groups was comparable (100.00 vs. 100.00%). In a subgroup analysis, the CADe system improved the PDR of the 6–9 mm polyps (18.04 vs. 13.85%, *p* < 0.05) and reduced the miss detection rate, especially at 10:00–12:00 am (12.5 vs. 39.81%, *p* < 0.001).

**Conclusion:**

The CADe system can potentially improve the sensitivity of endoscopists in detecting polyps, reduce the missed detection of polyps in colonoscopy, and reduce the risk of CRC.

**Registration:**

This clinical trial was registered with the Chinese Clinical Trial Registry (Trial Registration Number: ChiCTR2100041988).

**Clinical trial registration:**

website www.chictr.org.cn, identifier ChiCTR2100041988.

## Introduction

Colorectal cancer (CRC) is the third most common tumor worldwide and one of the leading causes of cancer-related deaths (1). CRC has a long incubation period with no obvious symptoms in the early stages; the majority of patients are not diagnosed until the disease has developed into an advanced stage. According to the 2020 colorectal cancer statistics, the 5-year survival rate of patients diagnosed with advanced colorectal cancer is only 12%. However, if CRC can be diagnosed and treated at an early stage, the 5-year survival rate of patients is more than 90% (2). Early diagnosis and subsequent treatment can effectively reduce mortality. Colonoscopy is the main method for screening for colorectal neoplasia and precancerous lesions. However, a meta-analysis of 43 tandem colonoscopies showed that up to 25% of colorectal neoplasia is missed after colonoscopy screening (3), which is the most relevant cause of post-colonoscopy colorectal cancer (4, 5).

Adenomatous polyps are the most common colorectal precancerous lesions, and they can be detected and removed by endoscopic procedures to prevent the occurrence and development of CRC. However, studies have shown that 45.9% of polyps are missed during colonoscopy (6). The primary cause of the high miss rate may be incomplete exposure of the colonic mucosal surface, and lesions may be hidden behind folds or food debris and not easily visualized. In addition, colonoscopy is technically challenging and demanding, requiring endoscopists to perform procedures and diagnoses. Less experienced endoscopists may easily ignore some lesions. Even experienced endoscopists may miss a non-obvious lesion due to a lack of concentration or fatigue (7, 8).

Artificial intelligence (AI) is an emerging science and technology that enables machines to simulate specific human thought processes and behaviors (9, 10). The advantage of AI lies in its ability to store more information and quickly parse the available data to perform complex visual perception tasks (11, 12). In 2016, deep learning algorithms were applied to various medical images, starting with diabetic retinopathy and pulmonary nodules. They also play an indispensable role in assisting doctors to diagnose diseases. (13–18). The original signal of the colonoscopy videos contains 25–30 frames per second, and a lesion may appear in only a few frames, which is one of the main reasons why endoscopists fail to detect lesions (19). The AI system is more sensitive and advantageous. It can process a large amount of image information in real time without fatigue and can detect subtle changes that are difficult for the human eye to distinguish. Some progress has been made in colonoscopy quality control and polyp detection (20–23). One study has shown that AI as an adjunct to colonoscopy can significantly improve the detection rate of colorectal neoplasia (24). Another meta-analysis showed that computer-aided detection (CADe) techniques based on AI significantly improved the adenoma detection rate over other techniques aimed at improving mucosal visualization, such as chromoendoscopy or narrow-band imaging (25). However, several key factors still need to be addressed before AI can be implemented clinically. One of them is that an AI model requires a sufficient number of annotated endoscopic images to achieve optimal performance and ensure model versatility, which may be challenging for AI in colorectal polyp detection due to the diversity of polyps and the need for expert annotation.

In this study, we exploited a novel CADe system (EndoAngel, Wuhan ENDOANGEL Medical Technology Co., Ltd.), which is capable of detecting colorectal polyps, recognizing the ileocecal region, and monitoring the speed of withdrawal during colonoscopy in real-time. This multicenter, prospective, randomized, controlled study aimed to evaluate the sensitivity and specificity of endoscopists with and without the assistance of the CADe system in a real-world clinical setting.

## Methods

### Study design and participants

This parallel, randomized, multi-center study was conducted at three Chinese endoscopy centers. Inclusion criteria were subjects aged 18–75 years with a need for colonoscopy diagnosis or screening, able to sign written informed consent, and with full legal capacity. Exclusion criteria were contraindications to colonoscopy (history of acute myocardial infarction within 6 months, severe hypohepatia, renal failure, and mental disorders), use of anticoagulants (aspirin, warfarin, etc.), known polyposis syndromes, familial polyposis, inflammatory bowel disease, known or highly suspected colorectal cancer, or colorectal surgery. Patients who were currently pregnant or participating in other clinical trials were also excluded. We obtained written informed consent from all patients before the colonoscopy. Our study followed the recommendations of the Consolidated Standards of Reporting Trials statement for reporting randomized controlled trials. This study was approved by the ethics committees of Ruijin Hospital of Shanghai Jiao Tong University School of Medicine, Beijing Cancer Hospital, and the Central Hospital of Wuhan. The study was registered under trial registration number ChiCTR2100041988 with the Chinese Clinical Trial Registry.^1^

### Randomization and masking

All eligible patients were randomly allocated (1:1) to receive either white light colonoscopy with the assistance of the CADe (experimental group) or without the assistance of the CADe (control group). We used computer-generated random numbers with no restrictions to determine each participant’s assignment. The randomization was done in blocks of four. The random assignment was blinded to the patients. The operating endoscopists were unaware of the overall study design and aims, but they were aware of the randomization status. Group allocations were concealed from data collectors and analysts.

### Procedures

A novel deep learning-based system (EndoAngel, Wuhan ENDOANGEL Medical Technology Co., Ltd.) was used in this prospective study. The system was developed on a deep learning framework with the help of endoscopists and modelers. The details of training, validation, and testing of this CADe are presented in the Supplementary material. The CADe was connected to the endoscopy processor, receiving the digital image as input and outputting a blue box only when a suspected polyp was captured in the field of view. The CADe system was installed on a separate computer system, and the output of the system appeared on a second monitor that was connected to the primary monitor via a serial digital interface cable. During the unassisted withdrawal, the second (CADe) monitor was turned off. During Artificial Intelligence (AI)-assisted withdrawal, the monitor was turned on.

The operating endoscopists were 21 staff members of the three endoscopy centers with colonoscopy experience of more than 1 year and a total volume of 100 colonoscopies. The endoscopes used in this study were manufactured by Olympus Optical. Before insertion, the operators were informed about the patient allocation.

Bowel preparation was assessed and graded on site by the endoscopists using the Boston Bowel Preparation Scale (BBPS); the BBPS score was recorded by an independent research assistant. BPPS from 0 to 3 were recorded in the three segments (descending colon, transverse colon, and ascending colon). After cecal intubation, the withdrawal time was measured in real time by the research assistant using a stopwatch. Cecal intubation was assessed by the endoscopists during the insertion procedure. The independent research assistant recorded whether polyps were detected and the location of polyps at each examination. If polyps were found, the routine diagnostic and treatment processes of each hospital were followed to decide whether to perform a polypectomy. The morphology of the colorectal polyps was determined according to the Paris Classification, which was divided into protruding lesions [>2.5 mm elevated above the mucosal layer: pedunculated (0-Ip), sessile (0-Is), or semi-pedunculated (0-Isp)], superficial lesions [slightly elevated by <2.5 mm (0-IIa), flat (0-IIb), or slightly depressed (0-IIc)], and laterally spreading tumors (LSTs).

The raw videos from each examination were screened and further analyzed to generate a gold standard for polyp detection. An independent evaluation group was established and was in charge of the process. Two experts with colonoscopy experience of over 5 years and a total volume of over 3,000 colonoscopies independently reviewed all the raw videos and labeled whether an examination was a positive one (with polyp detected) or a negative one (no polyp detected). The number, size, and morphology of the polyps were recorded by the two experts by reviewing the raw videos. In case of disagreement between the two experts, a third expert with colonoscopy experience of over 8 years and a total volume of over 5,000 colonoscopies would arbitrate and perform the final diagnosis. The diagnostic performance of the CADe system was also evaluated. A research assistant recorded the diagnostic results of the system. The performance of the CADe system was evaluated against the gold standard.

### Outcomes

The primary outcomes were endoscopist sensitivity and specificity with and without the assistance of the CADe system. Sensitivity = true positive/(true positive + false negative); specificity = true negative/(true negative + false positive). Secondary outcomes were diagnostic coincidence rate, false positive rate, false negative rate, positive predictive value (PPV), PPV = true positive/(true positive + false positive), negative predictive value (NPV), NPV = true negative/(true negative + false negative), positive likelihood ratio, negative likelihood ratio (NLR), balanced F1 score, polyp detection rate (PDR), BBPS score, and withdrawal time.

### Subgroup analysis of polyp detection

We further explored the PDR by stratifying the patients according to the location, size, and morphology of the polyps. Based on the gold standard, we evaluated the miss detection rate of endoscopists in the two groups stratified by the different time periods in a day.

### Statistical analysis

#### Sample size

The sample size was calculated based on the evaluation of the primary outcomes. This study used a co-primary outcome design, and both primary outcomes had to be fulfilled. We determined the specificity and sensitivity indices in this study based on the literature of similar artificial intelligence products (22, 26). The superiority and non-inferiority margins were set according to the Chinese guidelines for the design of medical device clinical trials and combined with the characteristics of the products in this study. As for the sensitivity of endoscopists to detect polyps, the sensitivity with the help of CADe was estimated to be 0.94, and the sensitivity without the help of CADe was estimated to be 0.80, with a superiority margin of 0.05; 217 polyps were identified in each group. According to the specificity of endoscopists for diagnosing polyps with or without the help of CADe, the CADe-assisted specificity was estimated to be 0.95, and the non-CADe-assisted specificity was estimated to be 0.95, with a non-inferiority margin of −0.05; 299 negative patients (no polyp detected) were required in each group. The PDR was estimated to be 45% based on our previous studies, so a total of 966 and 1,088 patients were needed. The larger sample size was obtained by considering a 20% dropout rate of a total of 1,360 patients invited in this trial.

#### Statistical analysis

Outcomes were analyzed in the FAS (full analysis set) and PPS (per-protocol set) populations. FAS refers to the set of eligible and withdrawn cases but excludes the excluded cases. Data from trials that were conducted and for which the primary outcome was available was entered into the FAS. The PPS included cases that met the study protocol, had good compliance, and completed all outcome evaluation indicators. The primary outcomes and metrics related to the miss detection rate were evaluated based on the gold standard generated by the expert panel. Other metrics were assessed based on the original data. Continuous variables were expressed as mean (SD) or median (IQR), according to their distribution, and categorical variables were expressed as n (%). Comparisons of proportions were done using the chi-square test and Fisher’s exact test. The Wilcoxon signed-rank test was used to compare the withdrawal time and BBPS score of the two groups. A negative binomial regression was used to compare the mean number of polyps in each patient. A two-tailed *p*-value of less than 0.05 was judged significant. Statistical analysis was performed using SAS 9.4.

## Results

### Patient enrollment and baseline data

**FIGURE 1:**
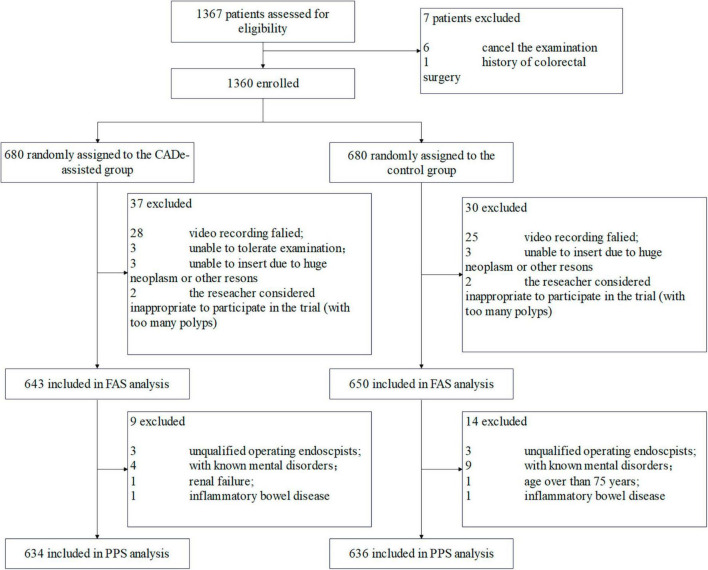
The flow diagram of the eligibility of the patients.

Between 17 January 2021 and 16 July 2021, a total of 1,367 consecutive patients were recruited and assessed for eligibility (Figure 1). In total, 7 patients were excluded. Therefore, 1,360 patients were randomly allocated to either the experimental group (with the assistance of CADe) or the control group (without the assistance of CADe). A total of 1,293 patients were finally included in the FAS analysis (643 in the experimental group and 650 in the control group). Another 23 patients were further excluded, and 1,270 patients were finally included in the PP analysis.

The baseline information is presented in Table 1. There was no statistically significant difference between the two groups with respect to demographic information (age, height, weight, or sex) or other baseline information.

**TABLE 1:** Baseline characteristics.

Items		CADe-assisted group	Control group	*p*-value
Age, year (SD)		52.87 (12.44)	53.20 (12.38)	0.663
**Sex**				
	Male subjects, *n* (%)	260 (40.44%)	291 (44.77%)	0.115
	Female subjects, *n* (%)	383 (59.56%)	359 (55.23%)	
Height, cm (SD)		164.95 (7.69)	165.43 (7.71)	0.286
Weight, kg (SD)		64.02 (11.78)	64.65 (12.25)	0.391
BMI		23.4	23.51	0.737
Family history, *n* (%)		109 (16.95%)	112 (17.23%)	0.894
Successful insertion, *n* (%)		643 (100.00%)	650 (100.00%)	NA
BBPS score		7.19 (1.32)	7.22 (1.37)	0.528
Withdrawal time, s (SD)		430.31 (111.06)	421.01 (100.83)	0.062

BMI, body mass index; BBPS, Boston bowel preparation score; CADe, computer-aided detection.

### Primary outcomes

#### Sensitivity comparison

The sensitivity of the endoscopists in detecting polyps with or without CADe was evaluated at the polyp level and the patient level, based on the gold standard generated by the expert panel. At the polyp level, a total of 1,011 and 1,110 polyps were detected in the experimental and control groups in the FAS analysis, respectively. The sensitivity of the endoscopists in the two groups was 84.97% (95% Confidence Interval [CI], 82.76–87.17%) and 72.07% (95% CI, 69.43–74.71%), the difference in sensitivity between the two groups was 12.89% (95% CI, 9.46–16.33%), with a difference in the lower limits of the 95% CI between the two groups of more than 5%, *p* < 0.001. In the PP analysis, a total of 993 and 1,093 polyps were detected in the experimental and control groups, respectively. The sensitivity of the endoscopists in the two groups was 84.99% (95% CI, 82.77–87.22%) and 72.37% (95% CI, 69.72–75.02%), *p* < 0.001. At the patient level, the sensitivity of the endoscopists between the two groups was 89.89% (96% CI, 86.85–92.94%) and 82.02% (96% CI, 78.28–85.76%) in the FAS analysis, and was 89.67% (96% CI, 86.56–92.78%) and 82.32% (95% CI, 78.57–86.08%) in the PP analysis. The result showed that in either FAS or PPS analysis, either in the polyp group or in the patient group, the sensitivity of the endoscopists was significantly improved with the assistance of the CADe system, and superiority validation was achieved. The results are shown in Table 2.

**TABLE 2:** Sensitivity and specificity of endoscopists with and without CADe.

Items			CADe-assisted group	Control group	*p*-value
Sensitivity evaluation	FAS	Positive, *n* (%)	859 (84.97%)	800 (72.07%)	<0.001
		Negative, *n* (%)	152 (15.03%)	310 (27.93%)	
		Total, *n*	1011	1110	
	PPS	Positive, *n* (%)	844 (84.99%)	791 (72.37%)	<0.001
		Negative, *n* (%)	149 (15.01%)	302 (27.63%)	
		Total, *n*	993 (0)	1093 (0)	
Specificity evaluation	FAS	Positive, *n* (%)	0 (0.00%)	0 (0.00%)	–
		Negative, *n* (%)	267 (100.00%)	244 (100.00%)	
		Total, *n*	267 (0)	244 (0)	
	PPS	Positive, *n* (%)	0 (0.00%)	0 (0.00%)	–
		Negative, *n* (%)	266 (100.00%)	240 (100.00%)	
		Total, *n*	266 (0)	240 (0)	

FAS, full analysis set; PPS, per-protocol set.

#### Specificity comparison

The specificity of the endoscopists in detecting polyps with or without CADe was evaluated at the patient level and based on FAS and PP analysis. According to the gold standard, a total of 267 and 244 negative patients in the experimental and control groups, respectively, were included in the FAS analysis; the specificity of the endoscopists in these two groups was 100.00% (95% CI, 98.63–100.00%) and 100.00% (95% CI, 98.50–100.00%), respectively. In the PP analysis, 266 and 240 negative patients were included; the specificity of endoscopists in these two groups was 100.00% (95% CI, 98.62–100.00%) and 100.00% (95% CI, 98.47–100.00%), respectively. The analysis showed that in either the FAS or PPS populations, the specificity of the endoscopists using the CADe system showed no significant difference; the difference in the lower limits of the 95% CI between the two groups was greater than −5%, and thus the non-inferiority validation was achieved. The results are shown in Table 2.

### Secondary outcomes

At the polyp level, the sensitivity of the CADe system in the FAS and PPS was 99.25% (95% CI, 98.78–99.57%) and 99.28% (95% CI, 98.82–99.60%), respectively. At the patient level, the sensitivity of the CADe was 100.00%. Compared to the gold standard, the diagnostic coincidence rate, false positive rate, false negative rate, positive predictive value (PPV), positive likelihood ratio, and balanced F1 score of the CADe system were 60.48% (57.81, 63.14%), 100.00% (99.28, 100.00%), 0.00% (0.00, 0.47%), 60.48% (57.81, 63.14%), 1, and 76.03% (FAS analysis). The results are presented in Table 3.

**TABLE 3:** Computer-aided detection (CADe) system performance.

Metrics	FAS	PPS
Sensitivity, % (95% CI)*	99.25% (98.78, 99.57%)	99.28% (98.82, 99.60%)
Specificity, % (95% CI)^	0.00% (0.00, 0.72%)	0.00% (0.00, 0.73%)
Diagnostic coincidence rate, % (95% CI)^	60.48% (57.81, 63.14%)	60.16% (57.46, 62.85%)
False positive rate, % (95% CI)^	100.00% (99.28, 100.00%)	100.00% (99.27, 100.00%)
False negative rate, % (95% CI)^	0.00% (0.00, 0.47%)	0.00% (0.00, 0.48%)
Positive predictive value, % (95% CI)^	60.48% (57.81, 63.14%)	60.16% (57.46, 62.85%)
Negative predictive value, % (95% CI)^	–	–
Positive likelihood ratio^	1	1
Negative likelihood ratio^	–	–
Balanced F1 score^	76.03%	75.79%

*Evaluated at the polyp level.

^Evaluated at the patient level. FAS, full analysis set; PPS, per-protocol set.

The BBPS score of the experimental and control groups had no significant difference in the FAS or PP analysis (7.19 [SD = 1.32] vs. 7.21 [SD = 1.37], *p* = 0.528; 7.19 [SD = 1.32] vs. 7.22 [SD = 1.37], *p* = 0.526). Details of the BBPS are presented in Tables 4, 5. The withdrawal times of these two groups were 430.31 (SD = 111.06) s and 421.01 (SD = 100.83) s (*p* = 0.062) in the FAS analysis and 430.23 (SD = 111.62) s and 421.38 (SD = 101.72) s (*p* = 0.074) in the PPS analysis, respectively, without significant difference. The PDR was 52.57% (338/643) and 51.23% (333/650) in the CADe-assisted group and the control group in the FAS analysis, respectively, without significant difference (*p* = 0.631). Similar results were found in the PPS analysis.

**TABLE 4:** Boston bowel preparation score (BBPS) in detail.

		FAS			PPS		
Location	Score	CADe-assisted group	Control group	Total	CADe-assisted group	Control group	Total
Cecum and ascending colon	0, *n* (%)	3 (0.47%)	4 (0.62%)	7 (0.54%)	3 (0.47%)	4 (0.63%)	7 (0.55%)
	1, *n* (%)	32 (4.98%)	51 (7.85%)	83 (6.42%)	32 (5.05%)	49 (7.70%)	81 (6.38%)
	2, *n* (%)	404 (62.83%)	378 (58.15%)	782 (60.48%)	395 (62.30%)	367 (57.70%)	762 (60.00%)
	3, *n* (%)	204 (31.73%)	217 (33.38%)	421 (32.56%)	204 (32.18%)	216 (33.96%)	420 (33.07%)
	Total	643 (0)	650 (0)	1293 (0)	634 (0)	636 (0)	1270 (0)
Transverse colon (liver curvature and spleen curvature)	0, *n* (%)	1 (0.16%)	3 (0.46%)	4 (0.31%)	1 (0.16%)	3 (0.47%)	4 (0.31%)
	1, *n* (%)	21 (3.27%)	22 (3.38%)	43 (3.33%)	21 (3.31%)	21 (3.30%)	42 (3.31%)
	2, *n* (%)	347 (53.97%)	334 (51.38%)	681 (52.67%)	341 (53.79%)	326 (51.26%)	667 (52.52%)
	3, *n* (%)	274 (42.61%)	291 (44.77%)	565 (43.70%)	271 (42.74%)	286 (44.97%)	557 (43.86%)
	Total	643 (0)	650 (0)	1293 (0)	634 (0)	636 (0)	1270 (0)
Descending colon to rectum	0, *n* (%)	1 (0.16%)	0 (0.00%)	1 (0.08%)	1 (0.16%)	0 (0.00%)	1 (0.08%)
	1, *n* (%)	30 (4.67%)	27 (4.15%)	57 (4.41%)	30 (4.73%)	26 (4.09%)	56 (4.41%)
	2, *n* (%)	233 (36.24%)	230 (35.38%)	463 (35.81%)	231 (36.44%)	228 (35.85%)	459 (36.14%)
	3, *n* (%)	379 (58.94%)	393 (60.46%)	772 (59.71%)	372 (58.68%)	382 (60.06%)	754 (59.37%)
	Total	643 (0)	650 (0)	1293 (0)	634 (0)	636 (0)	1270 (0)
Total score	0, *n* (%)	0 (0.00%)	0 (0.00%)	0 (0.00%)	0 (0.00%)	0 (0.00%)	0 (0.00%)
	1, *n* (%)	1 (0.16%)	2 (0.31%)	3 (0.23%)	1 (0.16%)	2 (0.31%)	3 (0.24%)
	2, *n* (%)	1 (0.16%)	1 (0.15%)	2 (0.15%)	1 (0.16%)	1 (0.16%)	2 (0.16%)
	3, *n* (%)	11 (1.71%)	14 (2.15%)	25 (1.93%)	11 (1.74%)	13 (2.04%)	24 (1.89%)
	4, *n* (%)	10 (1.56%)	10 (1.54%)	20 (1.55%)	10 (1.58%)	10 (1.57%)	20 (1.57%)
	5, *n* (%)	22 (3.42%)	19 (2.92%)	41 (3.17%)	22 (3.47%)	18 (2.83%)	40 (3.15%)
	6, *n* (%)	107 (16.64%)	106 (16.31%)	213 (16.47%)	105 (16.56%)	106 (16.67%)	211 (16.61%)
	7, *n* (%)	261 (40.59%)	245 (37.69%)	506 (39.13%)	257 (40.54%)	238 (37.42%)	495 (38.98%)
	8, *n* (%)	102 (15.86%)	122 (18.77%)	224 (17.32%)	99 (15.62%)	117 (18.40%)	216 (17.01%)
	9, *n* (%)	128 (19.91%)	131 (20.15%)	259 (20.03%)	128 (20.19%)	131 (20.60%)	259 (20.39%)
	Total	643 (0)	650 (0)	1293 (0)	634 (0)	636 (0)	1270 (0)

BBPS, Boston bowel preparation score; CADe, computer-aided detection; FAS, full analysis set; PPS, per-protocol set.

**TABLE 5:** Boston bowel preparation score (BBPS) evaluation and comparison.

		FAS			PPS		
**Location**	**Score**	**CADe-assisted group**	**Control group**	***p*-value**	**CADe-assisted group**	**Control group**	***p*-value**
		**(*n* = 643)**	**(*n* = 650)**		**(*n* = 634)**	**(*n* = 636)**	
Cecum and ascending colon	Mean (SD)	2.26 (0.57)	2.24 (0.62)	0.861	2.26 (0.57)	2.25 (0.62)	0.939
Transverse colon (liver curvature and spleen curvature)	Mean (SD)	2.39 (0.56)	2.40 (0.58)	0.517	2.39 (0.56)	2.41 (0.58)	0.490
Descending colon to rectum	Mean (SD)	2.54 (0.59)	2.56 (0.57)	0.533	2.54 (0.59)	2.56 (0.57)	0.553
Total	Mean (SD)	7.19 (1.32)	7.21 (1.37)	0.528	7.22 (1.37)	7.20 (1.34)	0.526

CADe, computer-aided detection; FAS, full analysis set; PPS, per-protocol set.

### Subgroup analysis of polyp detection

In both the FAS and PPS analyses, the PDR of polyps sized 6–9 mm showed a significant difference between the CADe-assisted group and the control group. The CADe system improved the PDR of 6–9 mm polyps. (18.04 vs. 13.85%, *p* < 0.05). The results are shown in Table 6. In the PPS analysis, the findings were similar.

**TABLE 6:** Subgroup analysis of polyp detection rate.

	FAS			PPS		
**Items**	**CADe-assisted group**	**Control group**	***p*-value**	**CADe-assisted group**	**Control group**	***p*-value**
	**(*n* = 643)**	**(*n* = 650)**		**(*n* = 634)**	**(*n* = 636)**	
**Location**						
Ascending colon, % (*n*)	17.42% (112)	15.54% (101)	0.362	16.88% (107)	15.57% (99)	0.526
Transverse colon, % (*n*)	19.75% (127)	18.15% (118)	0.464	19.87% (126)	18.24% (116)	0.458
Descending colon to rectum, % (*n*)	37.01% (238)	35.38% (230)	0.542	36.75% (233)	35.53% (226)	0.652
**Size**						
≤5 mm, % (*n*)	43.08% (277)	41.85% (272)	0.654	42.74% (271)	42.14% (268)	0.827
6–9 mm, % (*n*)	18.04% (116)	13.85% (90)	0.039	17.98% (114)	13.84% (88)	0.043
≥10 mm, % (*n*)	7.15% (46)	7.54% (49)	0.791	6.94% (44)	7.55% (48)	0.676
**Morphology**						
Flat, % (*n*)	48.99% (315)	46.15% (300)	0.307	48.58% (308)	46.23% (294)	0.401
Pedunculated, % (*n*)	3.11% (20)	2.92% (19)	0.844	3.15% (20)	2.99% (19)	0.863
Subpedicle, % (*n*)	7.93% (51)	6.92% (45)	0.489	7.57% (48)	6.92% (44)	0.654

CADe, computer-aided detection; FAS, full analysis set; PPS, per-protocol set.

In any time period of a day, the miss detection rate of the CADe-assisted group was significantly lower than that of the control group. In the 10:00–12:00 am period, the difference was much more significant (12.5 vs. 39.81%, *p* < 0.001). The results are shown in Table 7.

**TABLE 7:** Analysis of miss detection rate stratified by time of day.

	FAS			PPS		
**Time period**	**CADe-assisted group**	**Control group**	***p*-value**	**CADe-assisted group**	**Control group**	***p*-value**
8:00–10:00 am, % (*n*)	20% (19)	30.68% (27)	0.012	16.84% (16)	31.03% (27)	<0.001
10:00–12:00 am, % (*n*)	12.5% (16)	39.81% (43)	<0.001	12.5% (16)	39.25% (42)	<0.001
12:00–14:00 pm, % (*n*)	10.58% (11)	31.78% (34)	<0.001	10.68% (11)	30.84% (33)	<0.001
14:00–16:00 pm, % (*n*)	16.4% (71)	26.21% (114)	<0.001	15.81% (68)	26.45% (114)	<0.001

CADe, computer-aided detection; FAS, full analysis set. PPS, per-protocol set.

### Adverse effects

One case of adverse effect was found in the experimental group, which was slight bleeding during the procedure.

## Discussion

In this multi-center, parallel-controlled study, we evaluated the impact of endoscopists with and without the assistance of the CADe system on polyp detection. We statistically analyzed the data at the FAS and PPS levels. The results at both levels showed that the CADe could significantly improve the sensitivity of endoscopists to detecting polyps. It confirmed the effectiveness of the CADe in improving polyp detection during a colonoscopy screening. The specificity in both the experimental and control groups was 100%, indicating that the performance of the endoscopists using the CADe system was not inferior to that of the endoscopists in the control group without CADe.

The incidence and mortality of colorectal cancer remain high. Adenomatous polyps are important precursors of this type of malignancy. However, the polyp miss rate in colonoscopy is still high (7). This may be due to the small size of the early polyps and the slight mucosal changes that are difficult to detect with the naked eye. This is also susceptible to the patient’s bowel preparation and the physician’s level of experience and fatigue. In recent years, AI has made significant progress in the field of endoscopy (20). Compared with endoscopists, AI has a strong ability to identify tiny mucosal features, is less prone to fatigue, is not affected by the environment, etc. It can realize real-time localization and identification of colon polyps, which is expected to reduce the missed detection of polyps, thereby indirectly reducing the risk of CRC.

In our study, endoscopists using CADe achieved an absolute increase in sensitivity of 12% compared to the control group. There was no significant difference in PDR between the two groups. However, the PDR of medium-sized (6–9 mm) polyps was higher than that of the control group by more than 4.1% in both FAS and PPS analyses (*P* < 0.05). No statistical difference was found between the PDR of small polyps (≤5 mm) and large polyps (≥10 mm). Large polyps have a large mass and apparent mucosal lesions, so they may not be easily missed. For the PDR difference of small polyps (≤5 mm), it may require a larger sample size to verify the difference between the two groups. One of the main reasons for missed polyp detection is the difficulty in distinguishing suspicious lesions from normal colonic mucosa, which is also a cognitive challenge faced by endoscopists during colonoscopy. AI has unique advantages in identifying subtle features that can assist endoscopists in improving polyp detection.

We also counted the polyp miss rate (PMR) of different time periods of the day. The overall PMR of the experimental group was 12% lower than in the control group. The PMR in the experimental group was more than 10% lower than that of the control group at different times. Especially in the 10:00–12:00 period, which is usually considered the most tiring work period for endoscopists, the PMR decreased from 39.81 to 12.50% with the assistance of the CADe system. This confirmed that the AI system could offset part of the missed polyps due to fatigue.

In addition, in the experimental group, endoscopists’ specificity for polyps was not reduced by the potential effects of the CADe system, nor was the false positive rate for polyps increased. This suggests that the CADe system does not cause additional misdiagnosis by endoscopists. Interestingly, the average withdrawal time was 430.31 (SD = 111.06) s and 421.01 (SD = 100.83) s (*p* = 0.062) in the experimental and control groups, respectively, which both met the 6-min withdrawal time recommendation of international guidelines. The withdrawal time in the experimental group was longer; this may be due to the CADe system acting as a potential “supervisor” for the endoscopist. The lesion detection function with blue boxes shown on the colonoscopy monitor helped the endoscopist focus on suspicious lesions, thereby increasing the withdrawal time. This may also be one of the reasons for the increased sensitivity. However, the withdrawal times of the two groups showed no significant difference. Therefore, we are confident that the CADe system will improve the performance of the endoscopists without increasing their workload.

Most CRCs arise from traditional adenomas (including tubular adenomas, villous adenomas, and mixed tubular-villous adenomas) via the classic adenoma-carcinoma pathway (23). The detection and endoscopic resection of adenomas are essential for the prevention of colorectal cancer. Therefore, the adenoma detection rate (ADR) is an important criterion for assessing the quality of colonoscopy. However, recent studies have shown that 15–30% of sporadic CRCs develop through serrated lesions (24). Using the ADR as an evaluation index will lead to ignoring the serrated lesions. Polyps contain both traditional adenomas and serrated lesions. The improvement of the PDR is still significant for the long-term significance of preventing the occurrence of tumors in patients. Therefore, in this study, we paid great attention to the indicators related to the polyps from a more comprehensive perspective.

There are several limitations to our study. First, our study was conducted in three major digestive endoscopy centers in China, and the results may not have broad applicability. Further experiments can be conducted in hospitals in communities and remote, underdeveloped areas. In addition, we cannot rule out the subjective bias of endoscopists because the experiment could not be blinded to the operators. Operators tend to be more attentive when they learn that they are being observed, and the operation process will be more serious.

In conclusion, in this study, we evaluated the efficacy and safety of the CADe-assisted system in real-time colonoscopy in a natural clinical setting. The CADe system can potentially improve the sensitivity of endoscopists in detecting polyps, reduce the missed detection of polyps in colonoscopy, and reduce the risk of CRC.

## Data availability statement

The raw data supporting the conclusions of this article will be made available by the authors, without undue reservation.

## Ethics statement

The studies involving humans were approved by the Ruijin Hospital Ethics Committee, Shanghai Jiao Tong University, School of Medicine. The studies were conducted in accordance with the local legislation and institutional requirements. The participants provided their written informed consent to participate in this study.

## Author contributions

HZ: Methodology, Writing – original draft. QW: Methodology, Writing – original draft. JS: Data curation, Writing – original draft. JW: Data curation, Validation, Writing – original draft. LZ: Investigation, Writing – original draft. WC: Supervision, Writing – review and editing. DZ: Conceptualization, Supervision, Writing – review and editing.

## Footnotes

1
www.chictr.org.cn
