# Supplementary material for: A computer-aided system improves the performance of endoscopists in detecting colorectal polyps: a multi-center, randomized controlled trial
Source: Front Med (Lausanne). 2024 Jan 24;10:1341259. doi: 10.3389/fmed.2023.1341259 (PMC10847558; doi:10.3389/fmed.2023.1341259)
Supplement: Supplementary file 1 [file Data_Sheet_1.docx]

**Supplementary methods and materials**

**Training and testing of models**

We trained a YOLO model to recognize colon polyps, 8934 images of the colorectal polyp from 6015 patients, 9770 images of no polyps from 6015 patients were used for training and validation, 893 images of the colorectal polyp from 829 patients, and 977images of no polyp from 920 patients were used for the test. YOLO achieved an accuracy of 97.4% for recognizing colorectal polyps in test datasets. All the colonoscopy images and videos were in white light view, and from Renmin Hospital of Wuhan University. Instruments used in this study included gastroscopes from two vendors (Olympus Optical Co., Ltd., Tokyo, Japan. Fujifilm, Co., Kanagawa, Japan).

**Perceptual hash algorithm**

The perceptual hash algorithm is a widely used method in image retrieval, which produces a fingerprint character string for every image and then compares the fingerprints to identify similarities between images^1,2^. The difference hash (dHash), a branch of the perceptual hashing algorithm, was used as previously described^3^. Briefly, images were processed as follows: 1. Downsize images into 8×9 pixels to remove details in the image and only retain robust information such as the structure, light, and shade; 2. Convert downsized images into grayscale to simplify the comparison dimension; 3. Produce a 64-bit “hash fingerprint”, with “1” indicating the pixel intensity is increasing and “0” indicating it is decreasing in each line; 4. Calculate hamming distance, the number of positions where the corresponding bits are different between two strings of hash fingerprint: d(x,y)=∑x[i]⊕y[i]; 5. Calculate similarity score: Sim = [1-d(x,y)/64]×100. Sim indicates the similarity level between two images (the larger the value is, the more similar the two images are), and was used to detect previously seen images.

For monitoring real-time withdrawal speed, to mimic the human perceptual system that promotes longitudinal coherence over short-lived information, the weighted average method was used to analyze the similarity of ten successive frames according to the form: where Simi is the similarity score between frames n and n-(10-i). The deviation score was further calculated as: =100-, indicating the weighted average deviation level among ten successive images. The relationship between the value of and real-time withdrawal speed was explored using 5 stored colonoscopy videos without biopsy or therapy. The withdrawal process of the 5 videos was processed from 1 to 25 frames per second (fps). Taking the image processing interval as the independent variable and as the dependent variable, we performed linear regression using Pearson's correlation coefficient. It turned out that and image processing time interval were positively correlated, indicating that was positively-correlated with the withdrawal process.

1. Wen Z-k, Zhu W-z, Ouyang J, et al. A Robust and Discriminative Image Perceptual Hash Algorithm. 2010 Fourth International Conference on Genetic and Evolutionary Computing; 2010. p. 709-12.

2. Liao B, Xu J, Lv J, Zhou S. An Image Retrieval Method for Binary Images Based on DBN and Softmax Classifier. *IETE Tech Rev* 2015; **32**(4): 294-303.

3. Trends in Cyber-Physical Multi-Agent Systems. The PAAMS Collection - 15th International Conference, PAAMS 2017; 2018.
